# Effects of Faba Bean Hull Nanoparticles on Physical Properties, Protein and Lipid Oxidation, Colour Degradation, and Microbiological Stability of Burgers under Refrigerated Storage

**DOI:** 10.3390/antiox11050938

**Published:** 2022-05-10

**Authors:** Essam Mohamed Elsebaie, Ahmed Elmahdy, Eman S. El-Gezawy, Mohamed Reda Badr, Galila Ali Asker, Asmaa M. El-Gawish, Rowida Younis Essa

**Affiliations:** 1 Food Technology Department, Faculty of Agriculture, Kafrelsheikh University, Kafr El-Shiekh 33516, Egypt; rowida.eisa@agr.kfs.edu.eg; 2 Dairy Department, Faculty of Desert and Environmental Agriculture, Matrouh University, Fuka 10343, Egypt; ahmed_almahdi@mau.edu.eg; 3 Nutrition & Food Science Department, Faculty of Home Economics, Al-Azhar University, Tanta 31512, Egypt; emansobhy.el20@azhar.edu.eg (E.S.E.-G.); asmaael-gawish@azhar.edu.eg (A.M.E.-G.); 4 Food Science and Technology Department, Agriculture Faculty, Tanta University, Tanta 31512, Egypt; mohamed_badr@agr.tanta.edu.eg; 5 Food Science & Technology Department, Faculty of Home Economics, Al-Azhar University, Tanta 31512, Egypt; galilaalihamedaliasker@azhar.edu.eg

**Keywords:** FBH, FBH-NPs, burger, cooking, quality, TBARS, protein carbonyl content

## Abstract

The processing of faba beans generates great quantities of hulls, which are high in bioactive compounds with demonstrated radical-inhibiting properties. There is no research on the impact of using faba bean hull nanoparticles (FBH-NPs) to improve the quality and extend the shelf-life of beef products. Hence, the target of this investigation was to assess the inhibiting influence of adding FBH-NPs at two different concentrations (1 and 1.5%) on the physical attributes, lipid and protein oxidation, colour degradation, and microbiological safety of burgers during refrigerated storage (4 ± 1 °C/12 days). The FBH-NPs presented great phenolic content (103.14 ± 0.98 mg GAE/g dw) and antioxidant potential. The water holding capacity and cooking properties in burgers including FBH-NPs were improved during storage. The FBH-NPs significantly (p < 0.05) decreased the reduction rate of redness and lightness during the burger refrigerated storage and the FBH-NPs were more beneficial in preventing cold burger discolouration. In the FBH-NPs-treated burgers, peroxide values, TBARS, and protein carbonyl content were lower than in the control (up to 12 days). The microbiological load of burgers including FBH-NPs was lower than the load of the control during refrigerated storage. The findings revealed that FBH-NPs were more efficient in enhancing the cooking characteristics, retarding lipid or protein oxidation, preventing colour detrition and improving the microbial safety of burgers.

## 1. Introduction

Many nations throughout the world have seen a surge in the production and consumption of processed meat products such as patties, sausages, and burgers in recent years. Lipid oxidation is a primary source of quality degradation and shelf-life loss in foods containing fats such as meat products [1]. Food lipid oxidation can cause rancid odours and off-flavours, discoloration, drips and nutritional losses, the creation of hazardous components, and a reduction in shelf life [2]. As a result, sensory characteristics such as taste, odour, colour, and texture may suffer [3] while the use of artificial or natural antioxidants can reduce the rate and amount of oxidative damage. Artificial antioxidants like butylated hydroxy anisole (BHA) and butylated hydroxytoluene (BHT) have been used to reduce or inhibit oxidative reactions and preserve the sensory properties of meat products [4,5]; however, the consumption of these synthetic antioxidants may be related to toxicological aspects, such as DNA mutation and higher risks of developing neoplastic diseases [4]. Because of their safety and consumer acceptance, natural antioxidants are becoming increasingly popular for usage in foods. Thus, there is a growing search for natural products with antioxidant activity in order to replace synthetic antioxidants in meat products [6,7,8]. In this sense, by-products from plant food processing such as raspberry [9], pomegranate peel [10], pomegranate peel nanoparticles [11,12] and faba bean hulls [13] stand out, which are sources of natural antioxidants already reported in the manufacture of meat products. Waste products from the processing of legumes provide a practical and cost-efficient source of potent natural antioxidants that might replace artificial antioxidants [14].

The faba bean, also known as broad bean, fava bean, or *Vicia faba*, is a flowering plant that belongs to the *Fabaceae* family and *Vicia* genus [15]. The faba bean is a plant in the fabaceae family that has several uses. It is an important crop that is widely farmed in many regions of the world, particularly North Africa, on mixed rain-fed dry land or in agro-pastoral systems. Egypt was the world’s largest faba bean importer [16] and Egyptians consume a significant amount of faba beans, about 14 g per capita per day, which accounts for about 3 g of protein [17]. Güzel and Sayar [18] stated that the coat represents 14.28% of the weight of the seed. Large volumes of faba bean seed hulls (FBH) by-products are generated during the manufacture and processing of faba beans and they are either thrown away or turned into animal feed. Pyrogallol, e-vanillic acid, ellagic acid, and catechins are among the polyphenols found in the FBH by-products [16] and they have high antioxidant and antibacterial activities [19]. Nanotechnology has recently been used to develop new products with a variety of benefits for food manufacturing [20], including an increased shelf life and improved food quality and safety [21]. There is no research on the impact of using faba bean hull nanoparticles (FBH-NPs) to improve the quality and extend the shelf-life of beef products. The goal of this study was to evaluate (I) the antioxidant and antibacterial activities of FBH-NPs, as well as (II) the effects of adding FBH-NPs on the physical properties, protein and lipid oxidation, colour degradation, and microbiological stability of beef burgers throughout chilled storage.

## 2. Materials and Methods

### 2.1. FBH-NPs Preparation

FBH were bought from the feed market in the governorate of Kafr El-Sheikh, Egypt. FBH was converted to nanoparticle size using the method of Khataee et al. [22] with a slight modification. To create the FBH-NPs, the FBH was ground to a micro-particle size range of 100–150 µm using a Black & Decker grinder (Model BD54958, Black & Decker Inc, Minneapolis, MN USA) and crushed using a high-energy planetary ball-mill (Model PM2400, Kian Madan Pars Co, Tehran, Iran) at a speed of rotation of 320 rpm for 2 h. The ball-milling procedure was carried out at atmospheric conditions (26 °C at 1 atm) with a 10:1 ball-to-powder mass ratio. Finally, the average size of the homogeneous FBH-NPs was determined using a zetasizer (NS500, Malvern Panalytical, Worcestershire, UK). The obtained FBH-NPs (with an average size of 81 ± 4 nm) were then packed in a dark glass vial without oxygen and used in the tests immediately. To limit antioxidant loss, all operations used to make FBH-NPs were carried out under controlled settings.

### 2.2. Preparation of FBH-NPs Methanolic Extract

The FBH-NPs methanolic extract was made using a modified version of the process described by Barakat et al. [16]. Fifty millilitres of acidified methanol (1:99 *v*/*v*, HCl: MeOH) were combined with five grams of FBH-NPs. The homogenate was maintained at 2 °C in the dark for 12 h before being centrifuged at 660× *g* for 10 min and filtrated with Whatman paper No. 1. The filtrate solutions were kept at 20 °C until they were analysed.

### 2.3. FBH and FBH-NPs Methanolic Extract Analysis

The total phenolic (TP) concentration was determined using the method described by Ghasemzadeh et al. [23], and the findings were expressed as mg gallic acid equivalent (GAE)/g extract. Total flavonoid (TF) concentration was calculated as mg quercetin equivalent (QE)/g extract using the method described by Chang et al. [24].

Different in vitro techniques such as DPPH, ABTS, FRAP, and β-carotene bleaching were used to assess the antioxidant capacity of the FBH and FBH-NPs. The DPPH scavenging activity was established by the procedure of Turkmen et al. [25]. The ABTS test was carried out in accordance with Re et al. [26]. The β-carotene bleaching test was performed according to Boudjou et al. [27]. The ferric reducing antioxidant potential (FRAP) test was carried out according to Müller et al. [28].

### 2.4. Beef Burger Manufacture

Fresh beef (moisture 77.24%, fat 1.29%) and back fat (moisture 12.18%, fat 85.11%) were provided by the regional retail store (Kafr El-Sheikh, Egypt). Ten minutes after purchasing it, the meat was taken to the laboratory and placed inside an ice box. The beef flesh and back fat were ground twice in a meat grinder (Kenwood Meat Grinder MG470, Kenwood Company, Shanghai, China), first through an 8 mm plate and then through a 4 mm plate. The burger was made using the following ingredients: 85% minced beef, 13% minced fat, 1.3% salt, 0.65% seasoning mixture (cumin, black pepper, coriander, clove, and nutmeg) and 0.05% sodium tripolyphosphate. All of the ingredients were combined and stirred together for five minutes to produce a homogenous dough, which was then split into four groups. The first and second groups contained 1 and 1.5% of FBH-NPs, respectively. The third group had a BHT/BHA mixture in a 1:1 ratio at 0.2% as a control, while the fourth group had no antioxidants (control). Beef burgers weighing 72 ± 4 g were formed into 10 cm in diameter and 1 cm thick shapes using a press burger maker. The prepared burgers were placed into Styrofoam platters, wrapped with plastic film, and kept refrigerated at (4 ± 1 °C) for 12 days. The samples were tested every three days.

### 2.5. Burger Physical Properties

#### 2.5.1. pH Value and Water Holding Capacity (WHC)

The pH for the burgers was determined using a pH-metre (HAANA HI902 metre, Hanna Instruments, Smithfield, RI, USA) and the technique described by Abdelhakam et al. [3]. The WHC was calculated using the technique described by Yousefi et al. [29].

#### 2.5.2. Cooking Loss and Cooking Yield

To analyse the burger cooking attributes reported earlier by Essa and Elsebaie [1], the burgers were grilled on a White Whale electric grill (WA-BBQ01, White Whale Home Appliances, 10th of Ramadan City, Egypt) at about 180 °C for three minutes per side. The below equations were used to compute the cooking loss and cooking yield percentages:

$$\mathrm{Cooking}\;\mathrm{yield}\;(\%)=\frac{\mathrm{Cooking}\;\mathrm{weight}\;}{\;\mathrm{Raw}\;\mathrm{weight}\;}\;\times \;100$$

Cooking loss=100−Cooking yield 

### 2.6. Color Evaluation

#### 2.6.1. Surface Colour Measurement

The yellowness (b*), redness (a*), and lightness (L*) were measured objectively on the raw burger surface by the Hunter Lab Colorimeter apparatus (Colorflex, Broomfield, CO, USA) as previously stated by Essa and Elsebaie [30].

#### 2.6.2. Oxymyoglobin and Metmyoglobin Determination

The oxymyoglobin and metmyoglobin percentages in uncooked burgers were determined using the technique of Carlez et al. [31]. The below equations were utilised to calculate the oxymyoglobin and metmyoglobin percentages:

Oxymyoglobin(%)=[(0.882R-1.267R1)+(0.809R2-0.361) × 100]

Metmyoglobin(%)=[-2.541 R+0.777R1+0.800R2+1.098] × 100

where $R=\frac{\mathrm{Absorbance}\;\mathrm{at}\;572}{\mathrm{Absorbance}\;\mathrm{at}\;525}$, $R1=\frac{\mathrm{Absorbance}\;\mathrm{at}\;565}{\mathrm{Absorbance}\;\mathrm{at}\;525}$ $\mathrm{and}\;R2=\frac{\mathrm{Absorbance}\;\mathrm{at}\;545}{\mathrm{Absorbance}\;\mathrm{at}\;525}$.

### 2.7. Lipid Oxidation

#### 2.7.1. Peroxide Value Determination

The IDF technique was used to assess the peroxide value (PV) of the burger samples [32]. The data were provided in milliequivalents of peroxide kg^−1^ samples.

#### 2.7.2. Thiobarbituric Acid (TBARS) Determination

TBARS levels were measured in milligrams of malonaldehyde (MDA) per kilogram of specimen using spectrophotometric technique of Yoon et al. [33].

### 2.8. Total Protein Carbonyls Determination

Using 2,4-dinitrophenylhydrazine (DNPH), the protein oxidation was evaluated using the technique described by Mohamed et al. [34]. The carbonyl content was calculated as nmol of carbonyl per mg of protein at 370 nm using an extinction value of (21.0 nmol^−1^.cm^−1^).

### 2.9. Microbiological Analysis

A burger sample of 10 g was taken aseptically and transmitted to 90 mL of sterile peptone water (LAB104, LAB M, Edmunds, UK). For 2 min, the mixture was vortexed. After that, sequential decimal dilutions were made from the initial dilution and peptone water. To determine the total plate count (TPCs), psychrophilic bacteria, and lipolytic bacteria, 100 µL of each dilution was aseptically transmitted and spread on the surface of plate count agar medium (Difco Lab., Detroit, MI, USA). All agar plates were then incubated for 48 h at 37 °C in the case of TPCs and for 5 days at 7 °C for psychrophilic bacteria. In terms of lipolytic bacteria, the plates were incubated for 5 days at 37 °C. Subsequently, they were flooded with a 20% copper sulphate solution and the bacterial colonies with blue colour zones were enumerated [35].

## 3. Statistical Analysis

All treatments and analyses were performed in triplicate. All of the data were presented as mean±standard deviation (SD). Using SPSS software (v. 16.0 for Windows, SPSS Inc., Chicago, IL, USA), the data was analysed for variance (ANOVA) and the Duncan test to determine the significance (*p* < 0.05) amongst the treatments.

## 4. Results and Discussion

### 4.1. FBH-NPs Methanolic Extract Analysis

#### 4.1.1. TP and TF Content

As indicated in Table 1, the total phenolic compounds in the FBH and FBH-NPs methanolic extracts contained 82.31 and 103.14 mg GAE g^−1^ (on a dry basis), respectively. Hashemi and Ebrahimzadeh [36] found that the phenolic content of the methanolic extract of FBH varied from 78.6 to 110.3 mg GAE g^−1^. The difference in total phenolic content was related to the extraction technique used. The seed coats of other legumes likewise have higher phenolic content and antioxidant activity [37]. According to the current investigation, the TFC concentrations in the FBH and FBH-NPs methanolic extracts were 8.42 and 10.23 mg QE g^−1^, respectively. These findings are consistent with the information provided by Dawi et al. [38].

#### 4.1.2. Antioxidant Activities

The antioxidant capacity values of the FBH and FBH-NPs in vitro using several techniques such as DPPH, ABTS, FRAP, and beta-carotene/linoleic acid are presented in Table 2. By using the same experimental technique, the FBH antioxidant capacity value was dramatically (*p* < 0.05) less than that of the FBH-NPs. Methanolic extract of FBH-NPs presented lower antioxidant activity values than the artificial antioxidants when they were examined with the DPPH^•^ technique (IC50 = 112.51 ± 0.48 µg/mL) or with ABTS^•+^ (226.66 ± 1.31 µmol g^−1^ of Trolox). In the DPPH^•^ and ABTS^•+^ assays, alpha-tocopherol had the strongest antioxidant activity. The FBH-NPs had very low IC_50_ values (112.51 g/mL), indicating that they had potent antioxidant activity (Table 2). Hashemi and Ebrahimzadeh [36] obtained an IC_50_ value of (87.3 g mL^−1^) for FBH methanolic extract, which is lower than the values found in this investigation (Table 2).

During the β-carotene bleaching test, the β-carotene was oxidised, and as a result, smaller molecules were broken, the solution colour disappeared, and the yellowing discoloration of the β-carotene could be spectrophotometrically quantified [39]. The FBH and FBH-NPs lyophilized extracts demonstrated an 84.19 and 91.37% inhibition by the β-carotene bleaching test, respectively. When the FBH and FBH-NPs were compared to the positive controls (BHA/BHT mixture (1:1, *w*/*w*) and alpha-tocopherol) using the Tukey test, it was discovered that the FBH had the poorest antioxidant capacity, followed by the FBH-NPs (Table 2). The FBH-NPs, on the other hand, had an antioxidant activity of (387.09 ± 0.68 mol Fe^+2^ g^−1^) in the FRAP technique, whereas the industrial antioxidants (BHA/BHT mixture) had greater values. The purity of the commercial additives, which differs from the complexity of the examined FBH-NPs, might explain this disparity. Furthermore, due to worries about the negative health consequences of synthetic antioxidants, people increasingly prefer items with natural ingredients [40]. The antioxidant test findings clearly showed that FBH-NPs have a high antioxidant capacity, therefore, they may be employed as a natural antioxidant for preserving foods [27].

### 4.2. Effect of FBH-NPs on Burger Physical Properties

#### 4.2.1. pH and WHC Values

**Figure 1:**
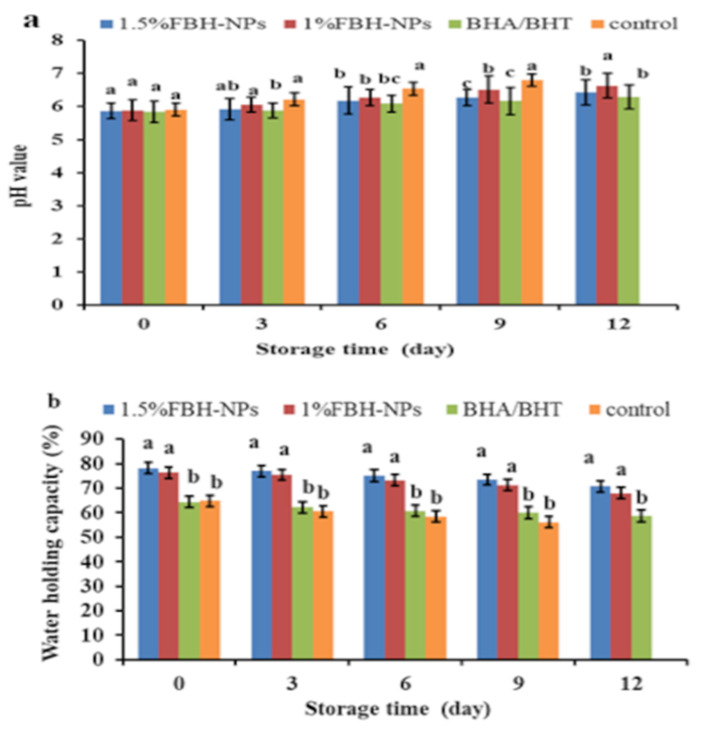
Changes in pH (**a**) and WHC (**b**) values of burgers incorporated with FBH-NPs and BHA/BHT during storage at 4 ± 1 °C. Error bars represent standard deviation, (*n* = 3). Different superscripts (a–c) lowercase letters indicate significant differences at *p* < 0.05 between treatments at each storage time.

Figure 1a shows the pH value as a burger quality parameter. At time zero, there were no significant variations (*p* < 0.05) in the pH of the various burger samples (containing BHA/BHT, 1% FBH-NPs, and 1.5% FBH-NPs) compared to the control. Furthermore, during storage at 4 ± 1°C, there were no significant variations in pH values (*p* < 0.05) between the burgers containing BHA/BHT and others containing 1.5% FBH-NPs. After nine cold-storage days, the pH of the burger control group climbed fast, reaching 6.80. Samples containing BHA/BHT and FBH-NPs at various concentrations, on the other hand, showed a small rise in pH throughout cold storage. After twelve storage days, the pH of the burger samples containing BHA/BHT was 6.30, while samples with 1% FBH-NPs and 1.5% FBH-NPs exhibited pH values of 6.63 and 6.43, respectively. The findings show an elevation in pH which may have been caused by the breakdown of nitrogenous substances via endogenous or microbiological enzymes [41].

One of the most essential indices for both manufacturers and customers is the meat’s WHC, which is defined as its capacity to retain all or a portion of its own and additional water [42]. Figure 1b depicts the WHC of a raw burger. The WHC was found to be significantly (*p* < 0.05) greater in samples containing FBH-NPs than in the control sample. There was an increase in WHC values as a consequence of the addition of more FBH-NPs. The high dietary fibre content of the FBH-NPs might be the most likely cause of the samples’ increased WHC. According to Kaya et al. [43], FBH includes 69.82 g dietary fibre per 100 g powder. According to Barakat [13], 1 g of FBH powder can contain 4.86 g of water, indicating that it might be employed as a thickening agent in the formulation of numerous foods. The same figure also shows that the WHC of all tested samples decreased significantly, whereas the highest WHC value (70.72%) at the end of the storage period was for the samples containing 1.5% FBH-NPs. This is owing to the active components included in the treatments’ capacity to preserve meat proteins from oxidation and degradation during storage. Soltanizadeh et al. [44] proved that increasing the content of natural additives protects the protein and improves the WHC. According to Viuda-Martos et al. [45], the rationale for the increase in WHC values is that these components helped to elevate the pH of the treated beef, which enhanced the WHC.

The favourable effect of FBH-NPs on the burger samples might be related to the water-binding characteristics of the FBH powder. The current findings are consistent with those reported by Embaby et al. [46].

#### 4.2.2. Cooking Yield and Cooking Loss

**Figure 2:**
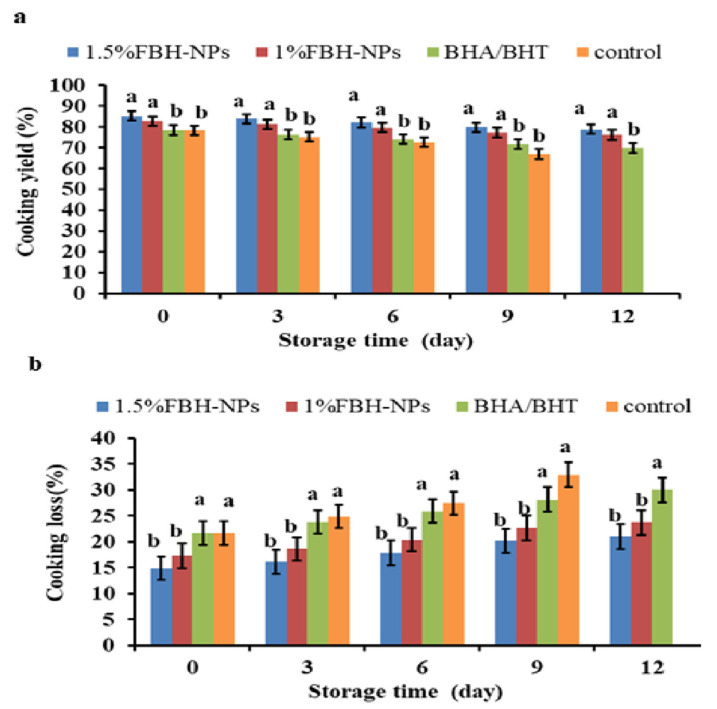
Changes in cooking yield (**a**) and cooking loss (**b**) percentages of burgers incorporated with FBH-NPs and BHA/BHT during storage at 4 ± 1 °C. Error bars represent standard deviation, (*n* = 3). Different superscripts lowercase letters indicate significant differences at *p* < 0.05 between treatments at each storage time.

As shown in Figure 2a, the cooking yield in the control sample was considerably lower (*p* < 0.05) than in the samples containing FBH-NPs. Cooking yield was considerably greater (*p* < 0.05) in the beef burgers containing FBH-NPs. When compared to other treatments, the beef burger that contained 1.5% FBH-NPs had the maximum cooking yield. This is most likely due to the FBH-NPs hydrocolloidal fibre’s ability to produce a three-dimensional matrix that holds not only water, but also fat added to the formula, preventing fat and water losses throughout the cooking process [47]. Fat was easier to extract from the control burgers after cooking, which was likely attributable to a low-density beef protein matrix and the great instability of fats. This is consistent with prior the investigation by Suman and Sharma [48]. The same figure also indicates that all the burger specimens’ cooking yield percentages were reduced as a consequence of extending the storage period from day 0 to 12. Nevertheless, burgers containing FBH-NPs appeared to have a higher cooking yield percentage than the other treatments. The control sample had the lowest cooking yield (67.04%), whereas samples with 1.5% FBH-NPs had the highest cooking yield (78.93%) after 12 days of storage.

However, as shown in Figure 2b, cooking loss percentages were greatly reduced (*p* < 0.05) in the beef burgers with FBH-NPs. Beef burgers containing 1.5% FBH-NPs gave the lowest cooking yield percentage in all tested specimens. In fact, the high cooking loss percentages were from the control group and burgers containing BHA/BHT. This might be attributable to the considerable loss of fat and moisture during the cooking process. In addition, there was an increase in the cooking loss for all tested samples throughout the whole storage period. Additionally, as shown in the same figure, cooking loss increased marginally in the burger samples made with FBH-NPs compared to the control sample over the cold storage time. Nevertheless, the burgers containing FBH-NPs appeared to have a lower cooking loss percentage than the other treatments. The samples with 1.5% FBH-NPs had the lowest cooking yield (21.07%) after 12 days of cold storage.

Generally, the cooking yield and cooking loss of beef burgers formed with FBH-NPs followed the same pattern as the WHC findings. The addition of FBH-NPs had a good influence on the cooking characteristics of the produced burger specimens. These findings might be attributed to the functional capabilities of FBH powder as a water-binding substance in general, rather than polyphenols specifically [16].

### 4.3. Effect of FBH-NPs on Burger Colour

Table 3 depicts the variations in instrumental colour values of uncooked burgers throughout the storage time. Except for the samples containing BHA/BHT, the hunter a* and hunter b* values declined considerably with extended storage time (*p* < 0.05).

However, over storage time, the hunter a* and hunter b* values in the burgers containing FBH-NPs were substantially greater than in the control sample (*p* < 0.05). The control sample showed the greatest hunter L* value (*p* < 0.05) when compared to the other treatments. As FBH-NPs were added to the burgers, they darkened, resulting in lower hunter L* values when compared with the control sample. Furthermore, increasing the concentration of FBH-NPs to 1.5% resulted in a steady decrease in hunter L* values (41.85). The dark hue of FBH caused a drop in hunter L* values for burgers reinforced with FBH-NPs. After storage, the burgers with the BHT/BHA mixture displayed a small decline in hunter L* value (40.87) when compared with the control specimen (41.10). In terms of changes in the hunter L* values over storage (zero to twelve days), all analysed samples showed a progressive reduction trend, suggesting increased darkening (Table 3).

Table 4 depicts the changes in oxymyoglobin and metmyoglobin percentages of the burgers as a function for cold storage time. The oxymyoglobin concentration of all treatments was reduced considerably over the course of 12 days (*p* < 0.05). The oxymyoglobin content of the burger-FBH-NPs was greater than that of the control. These findings revealed that the FBH-NPs significantly raised the oxymyoglobin content of burgers as compared to the control (*p* < 0.05).

The hunter a* value (Table 3) and oxymyoglobin percent (Table 4) in the burgers containing the BHT/BHA mixture were significantly (*p* < 0.05) greater than in the other treatments. Similarly, the proportion of metmyoglobin in all treatments was raised significantly with storage prolongation (*p* < 0.05). There was a significant change (*p* < 0.05) between the FBH-NPs-treated and control burgers. After 12 days of storage, the conversion of oxymyoglobin to metmyoglobin was reduced in the burgers containing 1% FBH-NPs and 1.5% FBH-NPs as compared with the control. There was a strong correlation (r = 0.97, *p* < 0.001) among burger redness (hunter a*) reduction, metmyoglobin production, and oxymyoglobin% decrease (data not presented).

The presence of oxymyoglobin is what gives minced beef its attractive red colour [49]. Metmyoglobin build-up and meat discolouration are mostly dependent on the existence of reducing mechanisms in meat and lipid oxidation during refrigerated storage [50]. Several researchers have related the development of oxidative responses to the decrease in redness in raw meats kept in refrigerated storage [51,52]. The Fe^2+^ in oxymyoglobin is known to be oxidised into Fe^3+^ in metmyoglobin via primary and secondary lipid oxidation products [50,53]. According to our results, FBH-NPs reduced the burger discolouration by decreasing the redness disappearance and oxymyoglobin concentration and preventing metmyoglobin rise. The phenolic components of FBH-NPs may have reduced the oxidation of lipids as well as oxymyoglobin in burgers.

### 4.4. Effect of FBH-NPs on Burger Lipid Oxidation

**Figure 3:**
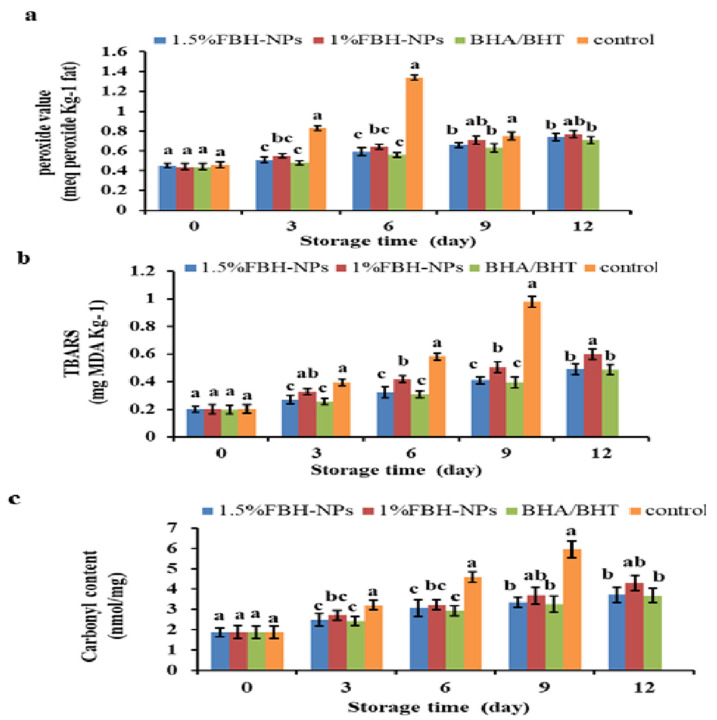
Changes in peroxide value (meq peroxide Kg^−1^ fat) (**a**), TBARS (mg MDA Kg^−1^) (**b**) and carbonyl content (nanomoles of carbonyl/milligram of protein) (**c**) percentages of burgers incorporated with FBH-NPs and BHA/BHT during storage at 4 ± 1 °C. Error bars represent standard deviation, (*n* = 3). Different superscripts lowercase letters indicate significant differences at *p* < 0.05 between treatments at each storage time.

Figure 3a shows that the addition of antioxidants and the storage duration had a significant (*p* < 0.05) influence on PV. The burger included polyphenol components from the spice mixture; the control group (no antioxidants were added) was tested.

The beginning PV ranged from 0.44 to 0.46 meq peroxide Kg^−1^ fat for all raw burger samples. On the sixth day, the PV of the control group surpassed the critical limit value, followed by a quick decline; however, the PV in burgers containing BHA/BHT, 1% FBH-NPs, or 1.5% FBH-NPs remained below the limit value until the 12th day of chilled storage. The results indicate that the control specimen displayed evident lipid-oxidation until the 6th day of storage and the highest PV as an indicator for the primary auto-oxidation termination. The PV reduced after 6 days of cold storage, probably owing to hydroperoxide breakdown to create secondary lipid oxidation products [54]. A small increase was seen when the BHA/BHT mixture was added to burgers after 9 days of cold storage. A modest increase in PV was seen in the sample containing 1.5% FBH-NPs. This shows that the pre-oxidation stage’s development and the breakdown of the peroxides produced were both moderate [55].

Figure 3b depicts the effect of antioxidant and storage period on malondialdehyde (MDA) content in the burger samples. Between the control and treated-burgers, there was a significant difference in TBARS (*p* < 0.05). During chilled storage, the lowest amount of TBARS was found in the burgers containing artificial antioxidants (BHA/BHT) and 1.5% FBH-NPs, while the highest value was found in the control specimen. The beginning TBARS value of all produced burger samples was ∼0.20 mg MDA/kg, which elevated fast in the control sample to 0.98 mg MDA/kg on the 9th day of storage, followed by 1% FBH-NPs (0.60 mg MDA/kg) on the 12th day, 1.5% FBH-NPs (0.492 mg MDA/kg) on the 12th day, and BHA/BHT (0.488 mg MDA/kg) on the 12th day.

The use of artificial and/or natural antioxidants considerably reduced the TBARS′ values even on the first day of storage. Furthermore, lipid oxidation-decrement was greatest in the 1.5% FBH-NPs burger when compared to the control or 1% FBH-NPs samples. Figure 3a,b shows that there was no significant (*p* < 0.05) difference in the PV and TBARS values between the burgers containing BHA/BHT and others containing 1.5% FBH-NPs under cold storage at 4 ± 1°C. FBH-NPs’ inhibitory impact on lipid oxidation is ascribed to their phenolic components, which exhibit antioxidant activity by preventing radical chain reactions during the oxidation reaction [56].

### 4.5. Effect of FBH-NPs on Burger Protein Degradation

As shown in Figure 3c, the protein carbonyls content raised dramatically (*p* < 0.05) in all stored burger specimens, while the highest content was achieved for the control group without antioxidants. After nine days of storage, the protein carbonyl concentration in the control specimen increased steadily from 1.87 to 5.94 nanomoles of carbonyl/milligram of protein. During chilled storage, the burgers containing 1.5% FBH-NPs had the smallest carbonyl concentration when compared to 1% LPP-NPs-treated. During storage, there were no significant variations in carbonyl content (*p* < 0.05) between burgers that were 1.5% FBH-NPs-treated and those with BHA/BHT. These findings show that FBH-NPs have a favourable influence on microbial growth suppression, particularly in proteolytic bacteria that promote protein degradation. The rise in the control sample carbonyl content observed after cold storage might be attributable to amino acid oxidation [57].

### 4.6. Microbiological Analysis

The TPCs, psychrotrophic count, and lipolytic bacteria count in the treated and control burgers raised significantly as the storage duration increased (Table 5). The rate of microbial growth increase was less in the BHA/BHT and FBH-NPs-treated burgers in comparison to the control. At day zero of storage, no psychrophilic bacteria or lipolytic bacteria were detected in any of the burger samples but they began to appear at the 3rd day of storage.

The inhibitory effect of bioactive and phenolic components found in FBH-NPs resulted in significantly lower TPCs, psychrotrophic, and thermophilic counts in all treated burgers when compared to the control at the end of the chilled storage duration. During the storage duration, there was no significant difference in the TPCs, psychrotrophic count, or lipolytic bacteria count among the burgers containing BHA/BHT and those containing 1.5% FBH-NPs. This could be related to the phenolics found in FBH-NPs, which have antioxidant and antimicrobial properties [58].

## 5. Conclusions

The phenolic content and antioxidant capacity of FBH-NPs were investigated in this work. FBH-NPs methanolic extract has a significant phenolic content and excellent antioxidant effects. During the chilled storage duration, the addition of FBH-NPs (1% or 1.5%) to the burgers, effectively enhanced their physical properties such as WHC and cooking yield as well as minimising cooking loss. Additionally, the FBH-NPs (1% or 1.5%) addition enhanced the colour stability and reduced the metmyoglobin content of the burgers compared to the other samples. Furthermore, the same levels of FBH-NPs were sufficient to inhibit protein and lipid oxidation throughout the storage days. Adding FBH-NPs (1% or 1.5%) to the burgers enhanced their microbiological stability under refrigerated storage by reducing the TPCs, psychrotrophic count, and lipolytic bacteria count compared with the other samples. Moreover, according to the obtained results, the addition of FBH-NPs to the burgers at 1.5% was the best concentration. Using FBH-NPs as natural antioxidants, as revealed in this study, might be a useful technique for improving burger quality.

## Figures and Tables

**Table 1:** Total phenols (mg GAE/g dw), total flavonoids (QE/g extract) contents of FBH and FBH-NPs.

Samples	Total Phenols (mg GAE/g dw)	Total Flavonoids (QE/g Extract)
**FBH**	82.31 ± 1.04 ^A^	8.42 ± 0.47 ^B^
**FBH-NPs**	103.14 ± 0.98 ^B^	10.23 ± 0.54 ^A^

FBH: Faba bean hulls; FBH-NPs: Faba bean hulls nanoparticles. Data are presented as mean ± SD. Means with different superscripts (A,B) uppercase letters in a column are significantly different at *p* < 0.05.

**Table 2:** Antioxidant activity of FBH and FBH-NPs.

Samples	DPPH (µg/mL) IC50	β-Carotene/Linoleic Acid (%)	ABTS (µmol/g de Trolox)	FRAP (µmol Fe^+2^/g)
**FBH**	123.65 ± 0.42 ^A^	84.19 ± 0.80 ^B^	202.28 ± 1.25 ^D^	345.80 ± 0.71 ^D^
**FBH-NPs**	112.51 ± 0.48 ^B^	91.37 ± 0.84 ^A^	226.66 ± 1.31 ^C^	387.09 ± 0.68 ^C^
**BHA/BHT**	93.46 ± 0.55 ^C^	65.87 ± 0.96 ^D^	4274.91 ± 66.28 ^B^	2513.95 ± 101.30 ^A^
**α-tocopherol**	62.08 ± 0.19 ^D^	71.24 ± 0.73 ^C^	4773.82 ± 91.15 ^A^	1325.76 c ± 24.08 ^B^

FBH: Faba bean hulls; FBH-NPs: Faba bean hulls nanoparticles. Data are presented as mean ± SD. Means with different superscripts uppercase letters in a column are significantly different at *p* < 0.05.

**Table 3:** Effect of adding FBH-NPs on colour parameters (L*, a* and b*) of burgers during refrigerated storage.

Treatments	0 day	3rd Day	6th Day	9th Day	12th Day
**a** *					
**Control**	15.23 ± 0.76 ^A, a^	12.60 ± 0.52 ^B, b^	10.47 ± 0.85 ^C, c^	8.36 ± 0.83 ^C, d^	Not determined
**BHA/BHT**	15.62 ± 0.91 ^A, a^	15.41 ± 0.66 ^A, a^	15.37 ± 0.92 ^A, a^	15.28 ± 0.90 ^A, a^	15.23 ± 0.96 ^A, a^
**1% FBH-NPs**	13.84 ± 0.84 ^B, a^	12.70 ± 0.78 ^B, b^	11.41 ± 0.73 ^B, c^	10.43 ± 1.08 ^B, d^	9.52 ± 0.99 ^B, e^
**1.5% FBH-NPs**	13.69 ± 0.85 ^B, a^	12.85 ± 0.84 ^B, b^	11.77 ± 0.91 ^B, c^	10.74 ± 1.10 ^B, d^	9.95 ± 0.73 ^B, e^
**b** *					
**Control**	14.10 ± 0.69 ^A, a^	12.71 ± 0.60 ^C, b^	11.59 ± 1.08 ^C, c^	9.22 ± 0.67 ^C, d^	Not determined
**BHA/BHT**	14.47 ± 1.01 ^A, a^	14.10 ± 0.73 ^A, a^	13.86 ± 0.55 ^A, a^	13.55 ± 0.74 ^A, a^	13.30 ± 0.69 ^A, a^
**1% FBH-NPs**	13.85 ± 0.58 ^B, a^	13.25 ± 0.92 ^B, a^	12.87 ± 0.87 ^B, b^	12.48 ± 0.98 ^B, b^	11.88 ± 1.00 ^B, c^
**1.5% FBH-NPs**	13.98 ± 0.77 ^B, a^	13.36 ± 1.10 ^B, a^	12.93 ± 0.64 ^B, b^	12.57 ± 0.59 ^B, b^	11.93 ± 0.76 ^B, c^
L*					
**Control**	43.28 ± 0.94 ^A, a^	42.74 ± 0.64 ^A, b^	42.17 ± 0.69 ^A, c^	41.10 ± 0.55 ^A, d^	Not determined
**BHA/BHT**	42.36 ± 0.75 ^B, a^	41.95 ± 1.18 ^A, B, a, b^	41.60 ± 0.84 ^B, b, c^	41.08 ± 0.80 ^A, c, d^	40.87 ± 0.67 ^A, d^
**1% FBH-NPs**	42.68 ± 0.89 ^B, a^	42.14 ± 0.56 ^A, a^	41.88 ± 0.95 ^B, a^	41.35 ± 1.07 ^A, a^	41.07 ± 0.99 ^A, a^
**1.5% FBH-NPs**	41.85 ± 1.03 ^C, a^	41.62 ± 0.75 ^B, a^	41.28 ± 0.72 ^B, a^	40.93 ± 0.61 ^A, B, a^	40.62 ± 0.58 ^A, a^

FBH: Faba bean hulls; FBH-NPs: Faba bean hulls nanoparticles. Data are presented as mean ± SD. Means with different superscripts uppercase letters in a column are significantly different at *p* < 0.05. Means with different superscripts lowercase letters in a row are significantly different at *p* < 0.05.

**Table 4:** Effect of adding FBH-NPs on oxymyoglobin and metmyoglobin content in burgers during refrigerated storage.

Treatments	0 Day	3rd Day	6th Day	9th Day	12th Day
**Oxymyoglobin (%)**					
**Control**	63.25 ± 0.42 ^B, a^	45.49 ± 0.72 ^D, b^	37.92 ± 0.66 ^D, c^	22.71 ± 0.84 ^D, d^	Not determined
**BHA/BHT**	64.78 ± 0.33 ^A, a^	56.80 ± 0.64 ^A, b^	53.49 ± 0.59 ^A, c^	51.88 ± 0.70 ^A, d^	50.32 ± 0.61 ^A, e^
**1% FBH-NPs**	58.23 ± 0.39 ^C, a^	50.54 ± 0.85 ^C, b^	46.63 ± 0.70 ^C, c^	37.64 ± 0.75 ^C, d^	31.07 ± 0.82 ^B, e^
**1.5% FBH-NPs**	56.14 ± 0.50 ^D, a^	51.97 ± 0.74 ^B, b^	48.02 ± 0.81 ^B, c^	41.87 ± 0.69 ^B, d^	38.17 ± 0.79 ^A, e^
**Metmyoglobin (%)**					
**Control**	14.78 ± 0.48 ^C, d^	27.37 ± 0.60 ^A, c^	31.58 ± 0.56 ^A, b^	52.87 ± 0.63 ^A, a^	Not determined
**BHA/BHT**	12.40 ± 0.44 ^D, e^	18.20 ± 0.73 ^D, d^	21.67 ± 0.49 ^D, c^	23.79 ± 0.54 ^D, b^	24.96 ± 0.74 ^B, a^
**1% FBH-NPs**	15.73 ± 0.72 ^B, e^	23.74 ± 0.59 ^B, d^	25.97 ± 0.66 ^B, c^	32.84 ± 0.70 ^B, b^	36.01 ± 0.41 ^A, a^
**1.5% FBH-NPs**	18.76 ± 0.81 ^A, e^	22.63 ± 0.62 ^C, d^	24.09 ± 0.57 ^C, c^	29.74 ± 0.62 ^C, b^	31.10 ± 0.55 ^B, a^

FBH: Faba bean hulls; FBH-NPs: Faba bean hulls nanoparticles. Data are presented as mean ± SD. Means with different superscripts uppercase letters in a column are significantly different at *p* < 0.05. Means with different superscripts lowercase letters in a row are significantly different at *p* < 0.05.

**Table 5:** Effect of adding FBH-NPs on microbiological load (Log CFU/g) in burgers during refrigerated storage.

Treatments	0 Day	3rd Day	6th Day	9th Day	12th Day
**Total plate count (Log CFU/g)**					
**Control**	3.51 ± 0.21 ^A, d^	4.92 ± 0.31 ^A, c^	6.80 ± 0.35 ^A, b^	7.43 ± 0.41 ^A, a^	Not determined
**BHA/BHT**	3.50 ± 0.33 ^A, c^	4.02 ± 0.28 ^C, b^	4.98 ± 0.27 ^C, a^	5.18 ± 0.38 ^B, a^	5.42 ± 0.33 ^A, a^
**1% FBH-NPs**	3.51 ± 0.27 ^A, d^	4.35 ± 0.26 ^B, c^	5.29 ± 0.24 ^B, b^	5.42 ± 0.32 ^B, a, b^	5.86 ± 0.41 ^A, a^
**1.5% FBH-NPs**	3.50 ± 0.29 ^A, c^	4.17 ± 0.30 ^C, b^	5.11 ± 0.36 ^C, a^	5.26 ± 0.29 ^B, a^	5.50 ± 0.37 ^A, a^
**Psychrotrophic count (Log CFU/g)**					
**Control**	Not detected	1.67 ± 0.26 ^A, c^	2.43 ± 0.42 ^A, b^	3.17 ± 0.36 ^A, a^	Not determined
**BHA/BHT**	Not detected	0.87 ± 0.15 ^C, c^	1.04 ± 0.33 ^C, b^	1.56 ± 0.35 ^C, a^	1.88 ± 0.30 ^B, a^
**1% FBH-NPs**	Not detected	1.12 ± 0.30 ^B, b^	1.44 ± 0.34 ^B, b^	1.80 ± 0.31 ^B, a^	2.25 ± 0.38 ^A, a^
**1.5% FBH-NPs**	Not detected	0.94 ± 0.28 ^C, b^	1.13 ± 0.29 ^C, b^	1.69 ± 0.39 ^A, B, a^	1.99 ± 0.44 ^A, a^
**Lipolytic count (Log CFU/g)**					
**Control**	Not detected	1.52 ± 0.33 ^A, c^	1.96 ± 0.29 ^A, b^	2.37 ± 0.47 ^A, a^	Not determined
**BHA/BHT**	Not detected	0.77 ± 0.36 ^C, c^	0.95 ± 0.32 ^C, b^	1.12 ± 0.22 ^C, a, b^	1.36 ± 0.27 ^B, a^
**1% FBH-NPs**	Not detected	0.96 ± 0.28 ^B, d^	1.23 ± 0.15 ^B, c^	1.61 ± 0.35 ^B, b^	2.07 ± 0.36 ^A, a^
**1.5% FBH-NPs**	Not detected	0.84 ± 0.20 ^C, c^	1.04 ± 0.26 ^C, b^	1.25 ± 0.28 ^C, a, b^	1.40 ± 0.19 ^B, a^

FBH: Faba bean hulls; FBH-NPs: Faba bean hulls nanoparticles. Data are presented as mean ± SD. Means with different superscripts uppercase letters in a column are significantly different at *p* < 0.05. Means with different superscripts lowercase letters in a row are significantly different at *p* < 0.05.

## Data Availability

The authors confirm that the data supporting the findings of this study are available within the article.
